# Transcriptome analysis reveals the defense mechanism of cotton against *Verticillium dahliae* in the presence of the biocontrol fungus *Chaetomium globosum* CEF-082

**DOI:** 10.1186/s12870-019-2221-0

**Published:** 2020-02-27

**Authors:** Yun Zhang, Na Yang, Lihong Zhao, Heqin Zhu, Canming Tang

**Affiliations:** 1 State Key Laboratory of Crop Genetics and Germplasm Enhancement, College of Agronomy, Nanjing Agricultural University, Nanjing, 210095 Jiangsu People’s Republic of China; 2 State Key Laboratory of Cotton Biology, Institute of Cotton Research of Chinese Academy of Agricultural Sciences, Anyang, 455000 Henan People’s Republic of China

**Keywords:** Transcriptome, Cotton, Verticillium wilt, Mechanism, Biocontrol fungus

## Abstract

**Background:**

Verticillium wilt of cotton is a serious soil-borne disease that causes a substantial reduction in cotton yields. A previous study showed that the endophytic fungus *Chaetomium globosum* CEF-082 could control Verticillium wilt of cotton, and induce a defense response in cotton plants. However, the comprehensive molecular mechanism governing this response is not yet clear.

**Results:**

To study the signalling mechanism induced by CEF-082, the transcriptome of cotton seedlings pretreated with CEF-082 was sequenced. The results revealed 5638 DEGs at 24 h post inoculation with CEF-082, and 2921 and 2153 DEGs at 12 and 48 h post inoculation with *Verticillium dahliae*, respectively. At 24 h post inoculation with CEF-082, KEGG enrichment analysis indicated that the DEGs were enriched mainly in the plant-pathogen interaction, MAPK signalling pathway-plant, flavonoid biosynthesis, and phenylpropanoid biosynthesis pathways. There were 1209 DEGs specifically induced only in cotton plants inoculated with *V. dahliae* in the presence of the biocontrol fungus CEF-082, and not when cotton plants were only inoculated with *V. dahliae*. GO analysis revealed that these DEGs were enriched mainly in the following terms: ROS metabolic process, H_2_O_2_ metabolic process, defense response, superoxide dismutase activity, and antioxidant activity. Moreover, many genes, such as *ERF*, *CNGC*, *FLS2*, *MYB*, *GST* and *CML*, that regulate crucial points in defense-related pathways were identified and may contribute to *V. dahliae* resistance in cotton. These results provide a basis for understanding the molecular mechanism by which the biocontrol fungus CEF-082 increases the resistance of cotton to Verticillium wilt.

**Conclusions:**

The results of this study showed that CEF-082 could regulate multiple metabolic pathways in cotton. After treatment with *V. dahliae*, the defense response of cotton plants preinoculated with CEF-082 was strengthened.

## Background

Cotton (*Gossypium* spp.) is an important economic crop species cultivated worldwide. Verticillium wilt of cotton is a serious vascular disease that detrimentally affects cotton yield and fiber quality [1] and is caused by the soil-borne fungus *Verticillium dahliae* Kleb. This disease can cause yellowing, wilting, defoliation, and ultimately death of cotton plants [2], and the pathogen is difficult to control because of its long-term survival as microsclerotia in the soil and its broad host range [3]. To date, no fungicide has been identified that can eliminate Verticillium wilt of upland cotton (*Gossypium hirsutum* L.) after plants have been infected [2, 4, 5].

At present, the use of biological control agents is a promising, more environmentally friendly strategy to control Verticillium wilt of cotton [6]. Numerous studies have shown that various biological control agents can suppress Verticillium wilt in different host species [7, 8]. Iturins mediate the defense response, and significantly activate *PR1*, *LOX*, and *PR10* at 24 h after *V. dahliae* infection [9]. The nonvolatile substances produced by CEF-818 (*Penicillium simplicissimum*), CEF-325 (*Fusarium solani*), CEF-714 (*Leptosphaeria* sp.), and CEF-642 (*Talaromyces flavus*) inhibit *V. dahliae* growth [10]. *Fusarium oxysporum* 47 (Fo47) reduced the symptoms of Verticillium wilt in pepper; the expression of three defense genes, *CABPR1*, *CACHI2* and *CASC1*, was upregulated in the roots [11]. *Bacillus subtilis* DZSY21 reduced the disease severity of southern corn leaf blight, and upregulated the expression level of *PDF1.2* [12]. Preinoculation of cauliflower with *Verticillium* Vt305 reduced symptom development and the colonization of plant tissues by *Verticillium longisporum* [13]. Various fungal and bacterial strains showed biocontrol activity against Verticillium wilt of olive. These microorganisms protect plants from the deleterious effects of the various pathogens, cause induced systemic resistance (ISR), compete for nutrients and colonization space, or promote plant growth through the production of phytohormones and the delivery of nutrients [14].

It has been reported that a series of immune reactions are induced in cotton plants infected with *V. dahliae*. In recent years, transcriptomic studies of the defense responses of plants infected with *V. dahliae* have become increasingly common, and several signal transduction pathways and key genes have been identified, including those involved in plant hormone signal transduction, plant-pathogen interaction, and phenylpropanoid-related and ubiquitin-mediated signals in cotton; additionally, these studies have investigated members of key regulatory gene families, such as receptor-like protein kinases (RLKs), WRKY transcription factors and cytochrome P450s (CYPs) [3]. The expression levels of *phenylalanine ammonia-lyase (PAL)*, *4-coumarate-CoA ligase (4CL)*, *cinnamyl alcohol dehydrogenase (CAD)*, *caffeoyl-CoA O-methyltransferase (CCoAOMT)*, and *caffeoyl O-methyltransgerase (COMT)* in the phenylalanine metabolism pathway have been shown to be upregulated in sea-island cotton [2]; the expression levels of 401 transcription factors (TFs), mainly in the MYB, bHLH, AP2-EREBP, NAC, and WRKY families, have been shown to be up- or downregulated in response to *V. dahliae* in *Arabidopsis thaliana* [15]; and genes encoding cyclic nucleotide gated channel (CNGC), respiratory burst oxidase homologue (RBOH), flagellin-sensitive 2 (FLS2), jasmonate ZIM domain-containing protein (JAZ), transcription factor MYC2, regulatory protein NPR1 and transcription factor TGA have been shown to be induced by *V. dahliae* in sunflower [16]. Several studies have investigated transcript levels in plants in response to biocontrol agents [17, 18].

In previous studies, we found that the endophytic fungus *Chaetomium globosum* CEF-082, which was isolated from upland cotton plants, suppressed the growth of *V. dahliae* and increased cotton resistance to Verticillium wilt [19]. However, the signalling mechanism induced by CEF-082 is unknown. Therefore, the purpose of this study was to reveal the molecular mechanism by which CEF-082 increased cotton resistance to Verticillium wilt via RNA sequence analysis.

## Results

### Control effect of CEF-082 on Verticillium wilt of cotton and H_2_O_2_ content

**Fig. 1:**
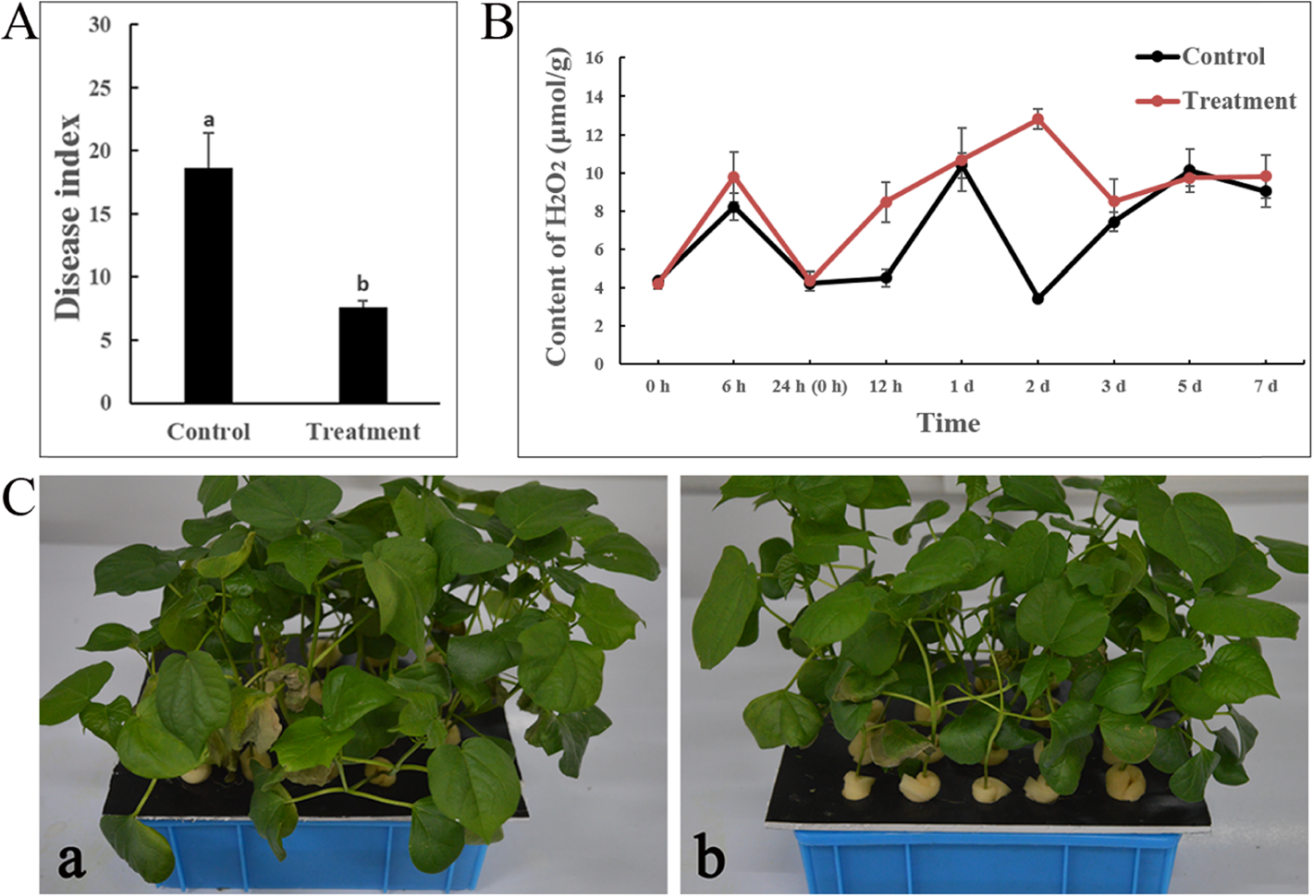
Disease index and symptoms of Verticillium wilt in cotton 14 d after *V. dahliae* inoculation. (A) The disease index. (B) The content of H_2_O_2_. (C) Symptoms of Verticillium wilt in cotton, a: water+*V. dahliae*, b: CEF-082 + *V. dahliae*. Bars represent SEs

The disease index was 18.61 in the control group (water+ *V. dahliae*) and 7.62 in the treatment group (CEF-082+ *V. dahliae*) 14 d after *V. dahliae* inoculation (Fig. 1A). The results showed that CEF-082 enhanced the resistance of cotton to Verticillium wilt, and the biocontrol effect was 59.1% (Fig. 1C).

The H_2_O_2_ content in the treatment group was higher than that in the control group throughout the majority of the duration of the experiment and lower than that in the control group at 5 dpi with *V. dahliae*. The H_2_O_2_ content in the treatment group was highest at 2 dpi (12.80 μmol/g), while the H_2_O_2_ content in the control group was highest at 1 dpi (10.38 μmol/g). The changes in the two groups were similar and were stable 5 d later (Fig. 1B).

### Verification of RNA-Seq analysis by qRT-PCR

**Fig. 2:**
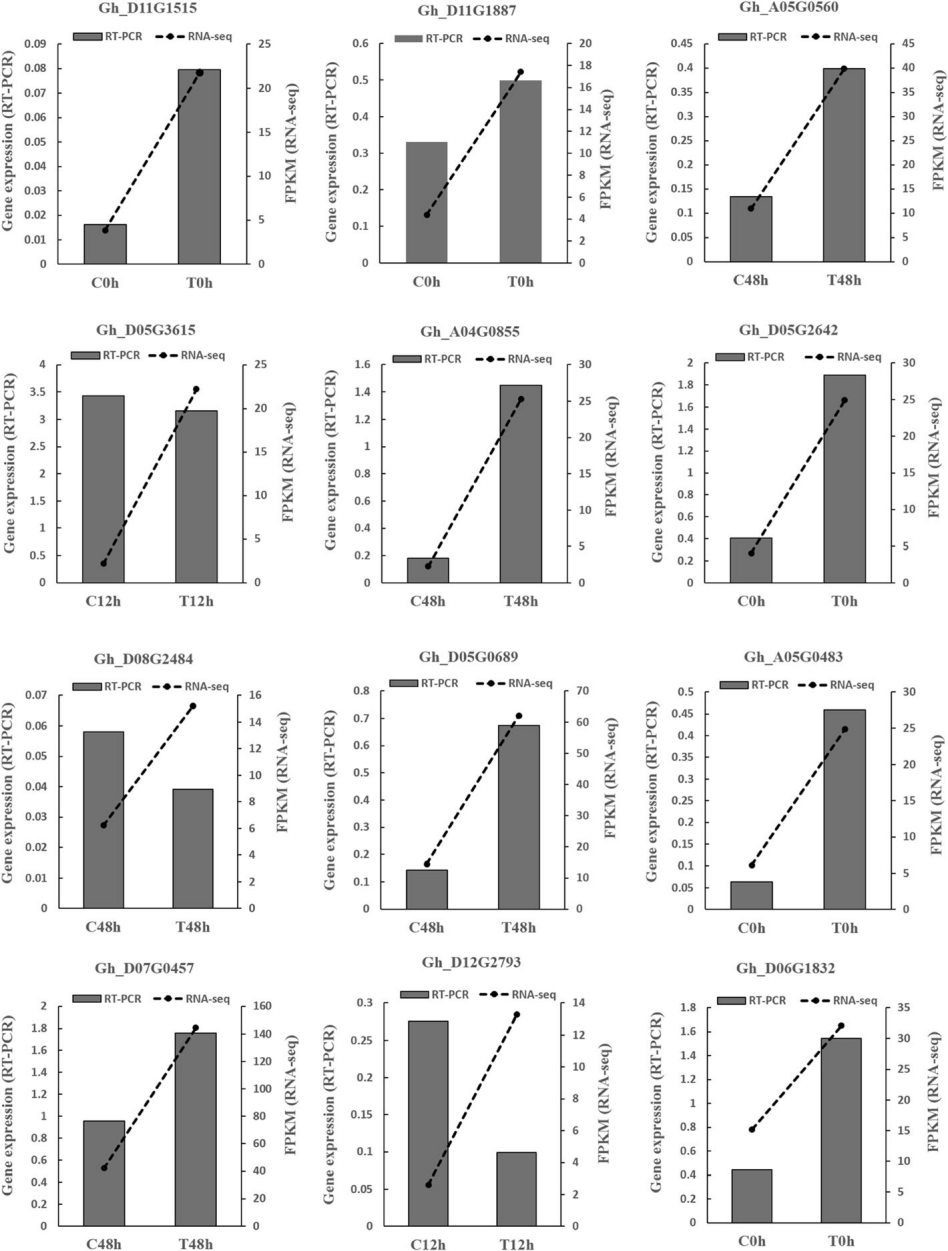
Comparison of the expression trends in the qRT-PCR and RNA-seq data. The gray bars represent the genes expression levels relative to that of the cotton *ubiquitin* gene, which was used as an internal control, to normalize the expression levels of the target genes. Dotted lines represent the mean FPKM

Twelve DEGs were randomly selected. The gene expression levels in the control and treatment groups were compared by qRT-PCR. The RNA-seq data showed that the expression of the 12 genes was upregulated at 0 h, 12 h or 48 h. The qRT-PCR results showed that the expression of nine of the 12 genes was upregulated, which was consistent with the results of their upregulated expression in the transcriptome; however, the expression of three genes was downregulated, which was inconsistent with their expression in the transcriptome, namely, *Gh_D12G2793*, *Gh_D08G2484* and *Gh_D05G3615* (Fig. 2). In addition, the levels of upregulation of 5 genes in the qRT-PCR data were lower than those in the RNA-seq data. The qRT-PCR data were up to 75% consistent with the transcriptome data.

### Functional annotation and enrichment analysis of the DEGs

The minimum correlation between the three replicates was 95.5% (Additional file 1: Figure S1). Principal component analysis (PCA) of 18 arrays (Additional file 2: Figure S2) was also used to compare the samples and to explore the dynamic changes in the cotton transcriptome after treatment with CEF-082 and *V. dahliae.*

The average clean reads of the 18 samples was 62.08 M. The lowest Q20 value of the clean reads was 97.93, and the lowest Q30 value was 90.06 (Additional file 9: Table S2). A total of 47,183 new transcripts were found, of which 7288 belonged to new protein-coding genes (Additional file 10: Table S3).

There were 3480 upregulated and 2158 downregulated DEGs at 0 h, 1716 upregulated and 1205 downregulated DEGs at 12 h, and 1524 upregulated and 629 downregulated DEGs at 48 h. The greatest number of DEGs was identified after inoculation with CEF-082 for 24 h. After inoculation with *V. dahliae*, the number of DEGs gradually decreased.

### DEGs induced by CEF-082 at 0 h without *V. dahliae* inoculation

**Fig. 3:**
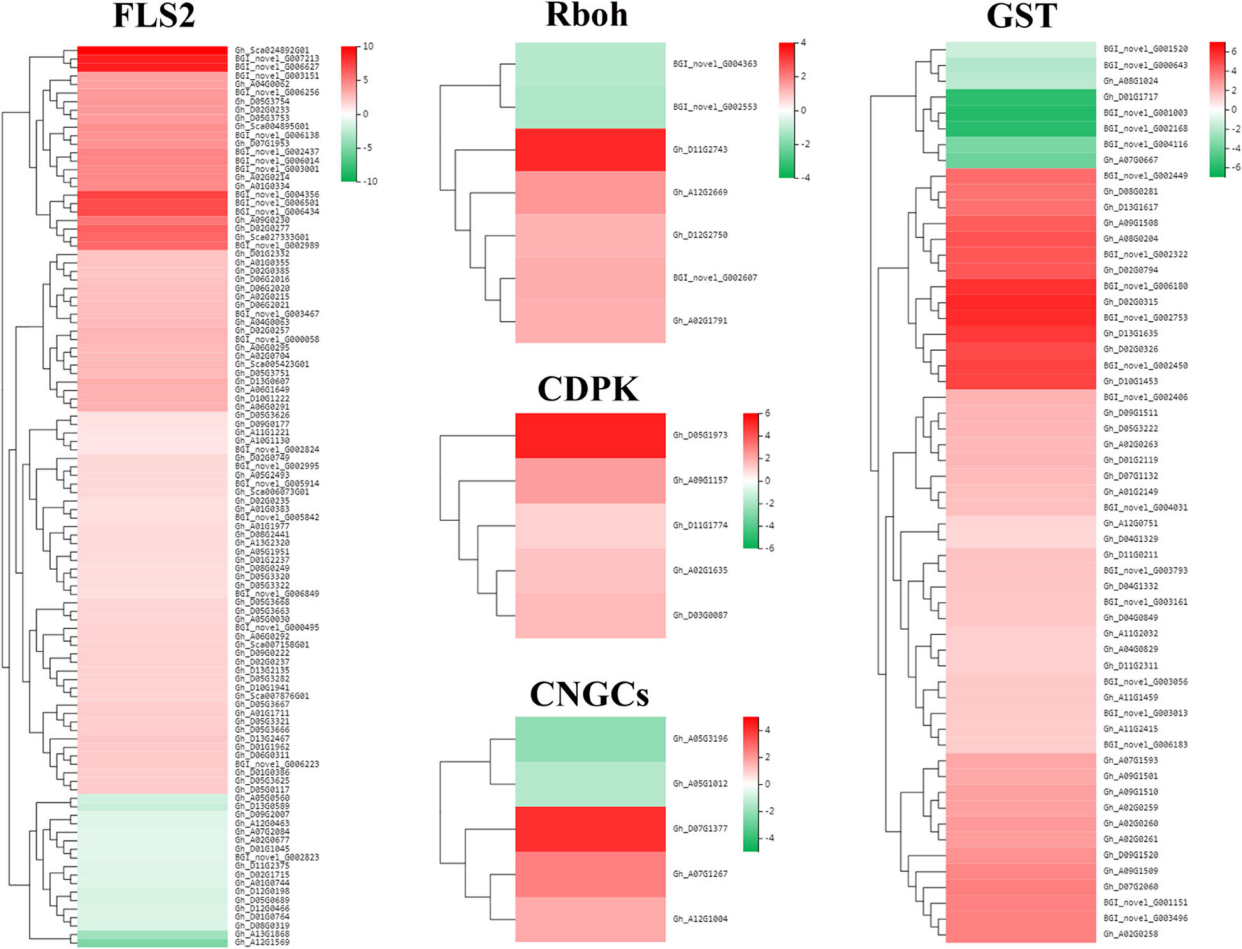
Expression levels of genes related to ROS and Ca^2+^. The red color represents upregulation, and the green represents downregulation

After inoculation with CEF-082 for 24 h (0 h), 5638 DEGs were identified, and KEGG pathway enrichment analysis revealed 15 significantly enriched pathways, including plant-pathogen interaction, MAPK signalling pathway-plant, flavonoid biosynthesis, phenylpropanoid biosynthesis, galactose metabolism, arachidonic acid metabolism, carotenoid biosynthesis, glutathione metabolism, sesquiterpenoid and triterpenoid biosynthesis, linoleic acid metabolism, other glycan degradation, glycosphingolipid biosynthesis - ganglio series, brassinosteroid biosynthesis, diterpenoid biosynthesis and sphingolipid metabolism (Q-value < 0.05) (Table 1). In the plant-pathogen interaction pathway, there were 106 *FLS2* genes, 88 upregulated and 18 downregulated; 7 *Rboh* genes, 5 upregulated and 2 downregulated; 5 upregulated *calcium-dependent protein kinase* (*CDPK*) genes; 5 *CNGC* genes, 3 upregulated and 2 downregulated; and 57 *glutathione S-transferase* (*GST*) genes in the glutathione metabolism pathway, of which 49 were upregulated and 8 downregulated (Fig. 3). These genes were related to the metabolism of reactive oxygen species (ROS) and Ca^2+^. In the MAPK signalling pathway-plant pathway, 304 DEGs regulated 30 crucial points related to ROS, Ca^2+^, abscisic acid (ABA), ethylene (ET), jasmonic acid (JA), H_2_O_2_ and FLS2. In the flavonoid biosynthesis pathway, the genes encoding chalcone synthase (CHS) and ferulate-5-hydroxylase (F5H) were induced. In the phenylpropanoid biosynthesis pathway, the key genes *PAL* and *4CL* were also induced.

**Table 1:** KEGG pathway enrichment of 5638 DEGs

Pathway ID	Pathway	Number of DEGs	*P*-value	Q-value
ko04626	Plant-pathogen interaction	376	2.57E-51	3.47E-49
ko04016	MAPK signalling pathway-plant	304	2.77E-25	1.87E-23
ko00941	Flavonoid biosynthesis	57	4.37E-08	1.97E-06
ko00940	Phenylpropanoid biosynthesis	135	1.80E-07	6.06E-06
ko00052	Galactose metabolism	88	6.14E-06	0.000166
ko00590	Arachidonic acid metabolism	34	2.09E-05	0.000469
ko00906	Carotenoid biosynthesis	39	0.000109	0.002102
ko00480	Glutathione metabolism	68	0.000138	0.002331
ko00909	Sesquiterpenoid and triterpenoid biosynthesis	21	0.000332	0.004976
ko00591	Linoleic acid metabolism	22	0.000788	0.010643
ko00511	Other glycan degradation	42	0.001074	0.013183
ko00604	Glycosphingolipid biosynthesis - ganglio series	27	0.001366	0.015371
ko00905	Brassinosteroid biosynthesis	15	0.001554	0.016135
ko00904	Diterpenoid biosynthesis	36	0.003500	0.033752
ko00600	Sphingolipid metabolism	44	0.004324	0.038919

Pathways with a corrected-*p* (Q-value) < 0.05 are shown

The GO enrichment analysis revealed that the 5638 genes were mainly enriched in 86 terms, including the intrinsic component of membrane, integral component of membrane, membrane part, membrane, catalytic activity, response to biotic stimulus, cell wall, oxidoreductase activity, defense response, response to stimulus, response to stress, and response to fungus (Q-value < 0.001), and the first 15 terms are listed in Table 2. Of the 16 genes in the response to fungus term, 15 were upregulated and 1 was downregulated. The GO classification showed that there were 18, 14 and 12 terms in biological process, cellular component and molecular function, respectively, and the KEGG classification indicated that the DEGs mainly belonged to the metabolism pathway (2856 DEGs).

**Table 2:** GO enrichment of 5638 DEGs

Term ID	Term	Number of DEGs	*P*-value	Q-value
GO:0031224	intrinsic component of membrane	1494	2.76E-26	6.24E-23
GO:0016021	integral component of membrane	1486	6.55E-25	7.42E-22
GO:0030246	carbohydrate binding	141	6.90E-22	5.20E-19
GO:0044425	membrane part	1506	2.47E-20	1.40E-17
GO:0009607	response to biotic stimulus	60	1.13E-18	5.14E-16
GO:0016020	membrane	1542	4.17E-18	1.57E-15
GO:0005576	extracellular region	152	8.49E-18	2.74E-15
GO:0001871	pattern binding	60	2.23E-17	5.60E-15
GO:0030247	polysaccharide binding	60	2.23E-17	5.60E-15
GO:0005618	cell wall	74	1.50E-14	3.08E-12
GO:0030312	external encapsulating structure	74	1.50E-14	3.08E-12
GO:0003824	catalytic activity	2165	3.11E-14	5.86E-12
GO:0051704	multi-organism process	59	5.08E-14	8.84E-12
GO:0044036	cell wall macromolecule metabolic process	45	9.14E-14	1.48E-11
GO:0071554	cell wall organization or biogenesis	119	1.43E-13	2.16E-11

Terms with a Q-value < 0.001 are shown

### DEGs coinduced by CEF-082 and *V. dahliae*

The 463 shared DEGs at 12 h and 48 h were significantly enriched in 6 KEGG pathways (Table 3). In the plant-pathogen interaction pathway, 29 DEGs regulated 8 crucial points, including CNGCs, calmodulin (CaM), FLS2, disease resistance protein RPS2 (RPS2), heat shock protein 90 kDa (HSP90), pto-interacting protein 1 (Pti1), disease resistance protein RPM1 (RPM1), and EIX receptor 1/2 (EIX1/2). In the phenylpropanoid biosynthesis pathway, 23 DEGs regulated 9 crucial points. In the flavonoid biosynthesis pathway, 12 DEGs regulated 8 crucial points. The enriched GO terms included terpenoid metabolic process, oxidoreductase activity, defense response, H_2_O_2_ metabolic process and ROS metabolic process terms.

**Table 3:** KEGG pathway enrichment of 463 DEGs

Pathway ID	Pathway	Number of DEGs	*P*-value	Q-value
ko00940	Phenylpropanoid biosynthesis	23	4.25E-06	0.000304
ko00941	Flavonoid biosynthesis	12	6.33E-06	0.000304
ko00052	Galactose metabolism	17	1.06E-05	0.000339
ko04626	Plant-pathogen interaction	29	0.000725	0.011595
ko00232	Caffeine metabolism	3	0.001058	0.014514
ko00909	Sesquiterpenoid and triterpenoid biosynthesis	5	0.001558	0.018699

Pathways with a Q-value < 0.05 are shown

### DEGs induced only in cotton inoculated with *V. dahliae* in the presence of CEF-082

A total of 1209 specific DEGs were identified at 12 h and 48 h, which were induced only in cotton plants inoculated with *V. dahliae* in the presence of CEF-082, but not when cotton plants were inoculated with *V. dahliae* only. The cluster thermogram showed the expression patterns of these genes at different stages (Additional file 3: Figure S3). KEGG classification showed that these DEGs mainly belonged to metabolism (672 DEGs) and were significantly enriched in 5 KEGG pathways, including flavonoid biosynthesis, indole alkaloid biosynthesis, MAPK signalling pathway-plant, plant-pathogen interaction, and phenylpropanoid biosynthesis (Table 4). GO classification showed that there were 14, 12 and 9 terms in the biological process, cellular component and molecular function, respectively. GO enrichment indicated that these DEGs were enriched in ROS metabolic process (14 DEGs), H_2_O_2_ metabolic process (12 DEGs), H_2_O_2_ catabolic process (12 DEGs), defense response (31 DEGs), superoxide dismutase activity (5 DEGs), antioxidant activity (19 DEGs), oxidoreductase activity, acting on superoxide radicals as acceptor (5 DEGs), cofactor binding (75 DEGs) and DNA binding (121 DEGs) (Additional file 4: Figure S4).

**Table 4:** KEGG pathway enrichment of 1209 DEGs

Pathway ID	Pathway	Number of DEGs	*P*-value	Q-value
ko00941	Flavonoid biosynthesis	19	0.000016	0.002007
ko00901	Indole alkaloid biosynthesis	14	0.000042	0.002610
ko04016	MAPK signalling pathway - plant	60	0.000474	0.019427
ko04626	Plant-pathogen interaction	59	0.001099	0.033788
ko00940	Phenylpropanoid biosynthesis	34	0.001932	0.047538

Pathways with a Q-value < 0.05 are shown

At 12 h and 48 h, 96 shared DEGs were obtained, which were induced only in cotton plants inoculated with *V. dahliae* in the presence of CEF-082, but not when cotton plants were inoculated with *V. dahliae* only (Additional file 5: Figure S5). KEGG analysis of the 96 DEGs indicated that they were mainly enriched in glutathione metabolism and flavonoid biosynthesis (Table 5). GO analysis showed that the DEGs were enriched in the terms superoxide dismutase activity, oxidoreductase activity, acting on superoxide radicals as acceptors, and antioxidant activity. Of the 96 DEGs, 9 encoded TFs and 20 encoded predicted PRGs (Additional file 11: Table S4).

**Table 5:** KEGG pathway enrichment of 96 DEGs

Pathway ID	Pathway	Number of DEGs	*P*-value	Q-value
ko00480	Glutathione metabolism	5	0.001184	0.035893
ko00941	Flavonoid biosynthesis	4	0.001496	0.035893

Pathways with a Q -value < 0.05 are shown

A protein-protein interaction network (Additional file 6: Figure S6) was constructed via the 96 DEGs shared between 12 h and 48 h and other genes interacting with them in cotton. Six hub genes were obtained, *Gh_A05G1020*, *Gh_D09G0858*, *BGI_novel_G004376*, *Gh_A08G0125*, *Gh_D07G1197* and *Gh_A05G3508*. Among them, *Gh_D07G1197* was annotated in the flavonoid biosynthesis pathway.

### Putative R genes and TFs involved in resistance to Verticillium wilt

**Fig. 4:**
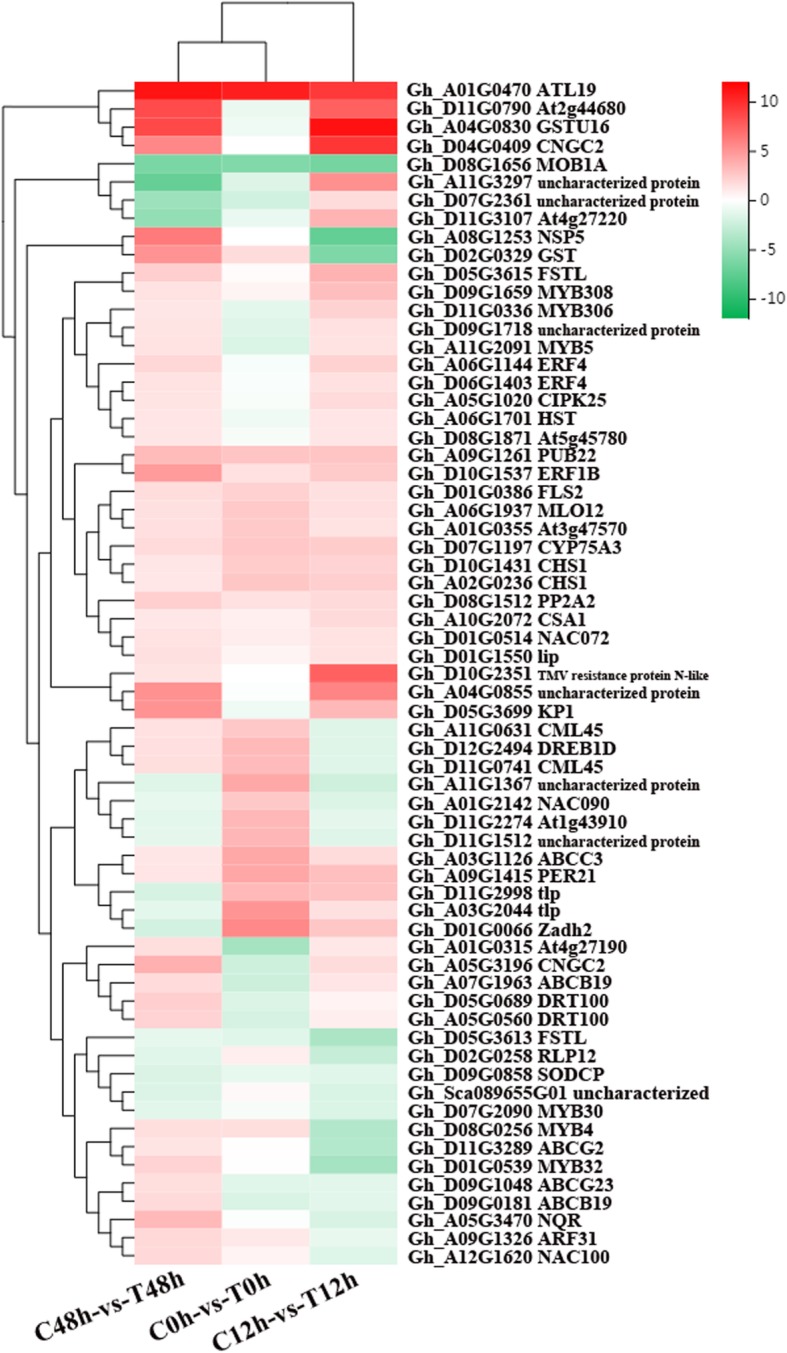
Clustering thermogram of putative R genes and genes encoding TFs

On the basis of the transcriptome analysis, a total of 65 candidate genes that may be related to the resistance of cotton to Verticillium wilt were identified, including 5 CNLs (whose members contain an NB-ARC domain), 3 CNs (members of the U-box domain-containing protein kinase family protein), 5 NLs (whose members contain an NBS-LRR domain), 7 RLPs (whose members contain an eLRR-TM-S/TPK domain), 7 Ns (whose members contain an NBS domain only), 9 TNLs (members of the TIR-NBS-LRR class), 6 Ts (members of NAC domain containing protein 17), 1 Mlo-like (a member of the Mlo-like resistant proteins) and 2 other types (which have resistance functions but do not fit the known classes). These genes mainly included a disease resistance protein, 2 probable calcium-binding protein (CML45), 3 ethylene-responsive transcription factor (ERF), 2 cyclic nucleotide-gated ion channel 2 (CNGC2), 5 MYB TFs and 2 GST (Tables 6, 7 and 8). A clustering thermogram of 65 genes (Fig. 4) showed that certain genes were upregulated at 0, 12 and 48 h; certain genes were downregulated at 0 h and upregulated at 12 and 48 h; and certain genes were downregulated at 0, 12 and 48 h.

**Table 6:** Predicted R genes induced by CEF-082 and *V. dahliae*

Transcript ID	Type	log2-Fold Change (12 h)	log2-Fold Change (48 h)	Nr Functional Annotation
Gh_A01G0315	CNL	1.037589939	1.4113336	disease resistance protein At4g27190-like, partial [*Gossypium hirsutum*]
Gh_A08G1253	CNL	−6.740709401	5.746950454	kelch repeat type 1 [*Corchorus capsularis*]
Gh_D02G0329	CNL	−5.561290274	4.673143293	probable glutathione S-transferase [*Gossypium hirsutum*]
Gh_D07G2361	CNL	1.502212669	−4.237409783	hypothetical protein B456_001G187600 [*Gossypium raimondii*]
Gh_D11G2274	CNL	−1.125013164	− 1.176243053	AAA-ATPase At1g43910-like [*Gossypium hirsutum*]
Gh_A04G0855	CN	5.289214165	4.734256842	uncharacterized protein LOC108457923 isoform X2 [*Gossypium arboreum*]
Gh_D09G1718	CN	1.293119787	1.144955552	uncharacterized protein LOC105800125 [*Gossypium raimondii*]
Gh_Sca089655G01	CN	−1.657997189	−1.535872284	uncharacterized protein LOC107949870 [*Gossypium hirsutum*]
Gh_A06G1937	Mlo-like	1.358973461	1.345179474	MLO-like protein 2 [*Gossypium hirsutum*]
Gh_A01G2142	NL	−1.561586712	−1.020990318	NAC domain-containing protein 90-like [*Gossypium arboreum*]
Gh_A09G1261	NL	2.484985295	2.969406864	E3 ubiquitin-protein ligase PUB23-like [*Gossypium hirsutum*]
Gh_A09G1326	NL	−1.010963189	1.6799656	B3 domain-containing protein At2g36080-like [*Gossypium arboreum*]
Gh_D08G1656	NL	−5.969729954	−5.677096641	MOB kinase activator-like 1A isoform X1 [*Gossypium hirsutum*]
Gh_D11G3107	NL	3.221799962	−4.647807823	uncharacterized protein LOC107925949 [*Gossypium hirsutum*]
Gh_A01G0470	N	8.54387182	10.19853857	putative RING-H2 finger protein ATL19 [*Gossypium hirsutum*]
Gh_A03G1126	N	1.517931636	1.090903245	putative ABC transporter C family member 15 [*Theobroma cacao*]
Gh_A07G1963	N	1.113205106	1.574240958	ABC transporter B family member 19-like isoform X1 [*Gossypium hirsutum*]
Gh_D09G0181	N	−1.254590611	1.59727426	ABC transporter B family member 19 [*Gossypium arboreum*]
Gh_D09G1048	N	−1.254965579	1.436429867	ABC transporter G family member 23-like [*Gossypium hirsutum*]
Gh_D11G0790	N	6.846798953	7.786264797	putative casein kinase II subunit beta-4 [*Gossypium hirsutum*]
Gh_D11G3289	N	−3.221611506	1.159392828	ABC transporter G family member 20-like [*Gossypium hirsutum*]
Gh_A01G0355	RLP	1.257686713	1.389731263	LRR receptor-like serine/threonine-protein kinase GSO1 [*Gossypium arboreum*]
Gh_D01G0066	RLP	2.472710792	−1.905542187	hypothetical protein B456_002G007800 [*Gossypium raimondii*]
Gh_D01G0386	RLP	1.338494948	1.470239195	probable LRR receptor-like serine/threonine-protein kinase At3g47570 isoform X1 [*Gossypium hirsutum*]

**Table 7:** Predicted R genes induced by CEF-082 and *V. dahliae*

Transcript ID	Type	log2-Fold Change (12 h)	log2-Fold Change (48 h)	Nr Functional Annotation
Gh_D05G3613	RLP	−3.636894741	−1.070068403	flavonol sulfotransferase-like [*Gossypium hirsutum*]
Gh_D05G3615	RLP	3.336220891	2.121111678	hypothetical protein B456_009G443300 [*Gossypium raimondii*]
Gh_D05G3699	RLP	3.056269729	4.673541612	kinesin KP1-like [*Gossypium raimondii*]
Gh_D08G1871	RLP	1.08210341	1.06909527	probable LRR receptor-like serine/threonine-protein kinase At1g34110 [*Gossypium hirsutum*]
Gh_A10G2072	TNL	1.586976576	1.014417675	TMV resistance protein N-like [*Gossypium hirsutum*]
Gh_A11G2091	TNL	1.199688858	1.260578514	transcription repressor MYB5-like [*Gossypium arboreum*]
Gh_D01G0539	TNL	−3.861003635	1.878860421	MYB-related protein 330 [*Gossypium hirsutum*]
Gh_D01G1550	TNL	1.22796154	1.351072869	lipase [*Corchorus capsularis*]
Gh_D07G2090	TNL	−1.58798514	−1.255994867	MYB transcription factor MYB30 [*Gossypium hirsutum*]
Gh_D08G0256	TNL	−3.281882012	1.421995876	transcription repressor MYB6-like [*Gossypium hirsutum*]
Gh_D09G1659	TNL	2.803942312	1.245838921	MYB-related protein 308-like [*Gossypium hirsutum*]
Gh_D10G2351	TNL	6.829614248	1.155493756	TMV resistance protein N-like isoform X2 [*Gossypium hirsutum*]
Gh_D11G0336	TNL	1.925007761	1.072893447	MYB-related protein 306 [*Gossypium hirsutum*]
Gh_A06G1144	T	1.922712186	1.745773435	ethylene-responsive transcription factor 4-like [*Gossypium hirsutum*]
Gh_A12G1620	T	−1.404115121	1.637338509	NAC domain-containing protein 100-like [*Gossypium hirsutum*]
Gh_D01G0514	T	1.231975072	1.260531662	NAC domain-containing protein 72-like [*Gossypium hirsutum*]
Gh_D06G1403	T	1.291258193	1.247681127	ethylene-responsive transcription factor 4-like [*Gossypium hirsutum*]
Gh_D10G1537	T	2.276313579	4.332088904	ethylene-responsive transcription factor 1B-like [*Gossypium raimondii*]
Gh_D12G2494	T	−1.419500468	1.382825786	putative dehydration responsive element binding protein [*Gossypium hirsutum*]
Gh_A03G2044	Other	1.343265394	−1.171383404	thaumatin-like protein [*Gossypium arboreum*]
Gh_D11G2998	Other	2.626616578	−1.757148487	thaumatin-like protein isoform X1 [*Gossypium hirsutum*]
Gh_A02G0236	/	2.076793222	1.040351538	chalcone synthase [*Vaccinium ashei*]
Gh_A04G0830	/	10.27309684	7.911807354	glutathione S-transferase U16-like [Gossypium hirsutum]

**Table 8:** Predicted R genes induced by CEF-082 and *V. dahliae*

Transcript ID	Type	log2-Fold Change (12 h)	log2-Fold Change (48 h)	Nr Functional Annotation
Gh_A05G0560	/	0.753709773	1.881639236	DNA-damage-repair/toleration protein DRT100-like [*Gossypium raimondii*]
Gh_A05G1020	/	1.557304461	1.177105999	CBL-interacting serine/threonine-protein kinase 25-like [*Gossypium hirsutum*]
Gh_A05G3196	/	1.49233142	3.400801654	cyclic nucleotide-gated ion channel 2-like [*Gossypium arboreum*]
Gh_A05G3470	/	−1.679248485	3.0042548	NADPH:quinone oxidoreductase-like [*Gossypium arboreum*]
Gh_A06G1701	/	1.11748676	1.125924709	shikimate O-hydroxycinnamoyltransferase-like [*Gossypium arboreum*]
Gh_A09G1415	/	2.763055805	1.138512634	peroxidase 21-like [*Gossypium hirsutum*]
Gh_A11G0631	/	−1.427211837	1.313850285	probable calcium-binding protein CML45 [*Gossypium hirsutum*]
Gh_A11G1367	/	−2.131571879	−1.430486114	uncharacterized protein LOC105803388 [*Gossypium raimondii*]
Gh_A11G3297	/	4.769980778	−6.621901362	uncharacterized protein LOC107935227 [*Gossypium hirsutum*]
Gh_D02G0258	/	−2.480311701	−1.300029512	receptor-like protein 12 [*Gossypium hirsutum*]
Gh_D04G0409	/	8.681445478	5.183990265	cyclic nucleotide-gated ion channel 2-like [*Gossypium hirsutum*]
Gh_D05G0689	/	0.530244279	2.104072295	DNA-damage-repair/toleration protein DRT100 [*Theobroma cacao*]
Gh_D07G1197	/	2.197263059	1.546938795	flavonoid 3′,5′-hydroxylase 2-like [*Gossypium hirsutum*]
Gh_D08G1512	/	1.61343892	2.05940005	hypothetical protein B456_002G144600 [*Gossypium raimondii*]
Gh_D09G0858	/	−1.30017514	−1.614570284	hypothetical protein B456_006G104900, partial [*Gossypium raimondii*]
Gh_D10G1431	/	1.879317791	1.074465933	chalcone synthase [*Kandelia candel*]
Gh_D11G0741	/	−1.323988617	1.425564743	probable calcium-binding protein CML45 [*Gossypium hirsutum*]
Gh_D11G1512	/	−1.351190116	−1.111728188	uncharacterized protein LOC105803388 [*Gossypium raimondii*]
Gh_D11G3107	/	3.221799962	−4.647807823	uncharacterized protein LOC107925949 [*Gossypium hirsutum*]

## Discussion

The number of DEGs identified at 12 h and 48 h was lower than that identified at 0 h. The number of DEGs may have decreased in these cases because the plants were infected with *V. dahliae* and began to respond defensively. The DEGs between the CEF-082 treatment and CEF-082+ *V. dahliae* treatment, were enriched mainly in 5 signalling pathways: plant-pathogen interaction, MAPK signalling pathway-plant, flavonoid biosynthesis, phenylpropanoid biosynthesis, and glutathione metabolism. The pathways of plant-pathogen interaction and flavonoid biosynthesis were also induced in sunflower plants infected with *V. dahliae* [16], and the results were also consistent with those of Tan [20], who reported that most DEGs in tomato were associated with phenylpropanoid metabolism and plant-pathogen interaction pathways. However, the glutathione metabolism pathway has rarely been reported in the transcriptome of cotton plants treated with *V. dahliae*.

It is clear that plant responses to biotic or abiotic stress depend on interactions among several signalling pathways, including those mediated by JA, ET, salicylic acid (SA) or ABA [21, 22]. Morán-Diez et al. [17] found SA- and JA-related DEGs were downregulated in *A. thaliana* after 24 h of incubation in the presence of *Trichoderma harzianum* T34. A set of DEGs influenced by JA or ET was induced upon pathogen attack when *A. thaliana* was previously colonized by a photosynthetic *Bradyrhizobium* sp. strain, ORS278 [18]. DEGs related to ET, SA, JA, brassinosteroid (BR) and cytokinin were upregulated or downregulated upon *V. dahliae* infection in cotton [3]. In this study, we also found that DEGs in ABA, auxin and gibberellin were significantly induced not only after treatment with CEF-082 but also after inoculation with *V. dahliae*. In addition, DEGs related to JA, ET, SA, BR and cytokinin were induced in cotton plants treated only with CEF-082. The 8 plant hormones were also induced after infection with *V. dahliae* in sunflower [16]. The responses of the *A. thaliana* auxin receptors TIR1, AFB1 and AFB3 and auxin transporter AXR4 were impaired upon infection with *V. dahliae* [23]. Therefore, both CEF-082 and *V. dahliae* can induce changes in hormones.

Previously, it was shown that after plants were infected with pathogens, the FLS2 pattern recognition receptors recognized pathogens, and the hypersensitive response (HR) was activated through ROS, JA, WRKYs and the NO signalling pathways [24, 25] and mediated by CNGC, RBOH, CaM/CML and FLS2 [26–28]. These results are consistent with the results of this study. In this study, 24 h after treatment with CEF-082, the DEGs of FLS2, Rboh, CDPK, CNGCs and GST in the plants were also upregulated or downregulated to varying degrees (Fig. 3). In addition, most of the genes encoding peroxidase (POD), superoxide dismutase (SOD), and catalase (CAT) were also upregulated. These genes were related to the accumulation of ROS. Forty-eight hours after treatment with *V. dahliae*, the genes encoding CNGC, CaM/CML and FLS2 were upregulated. However, in this study, the NO signalling pathway was not induced.

**Fig. 5:**
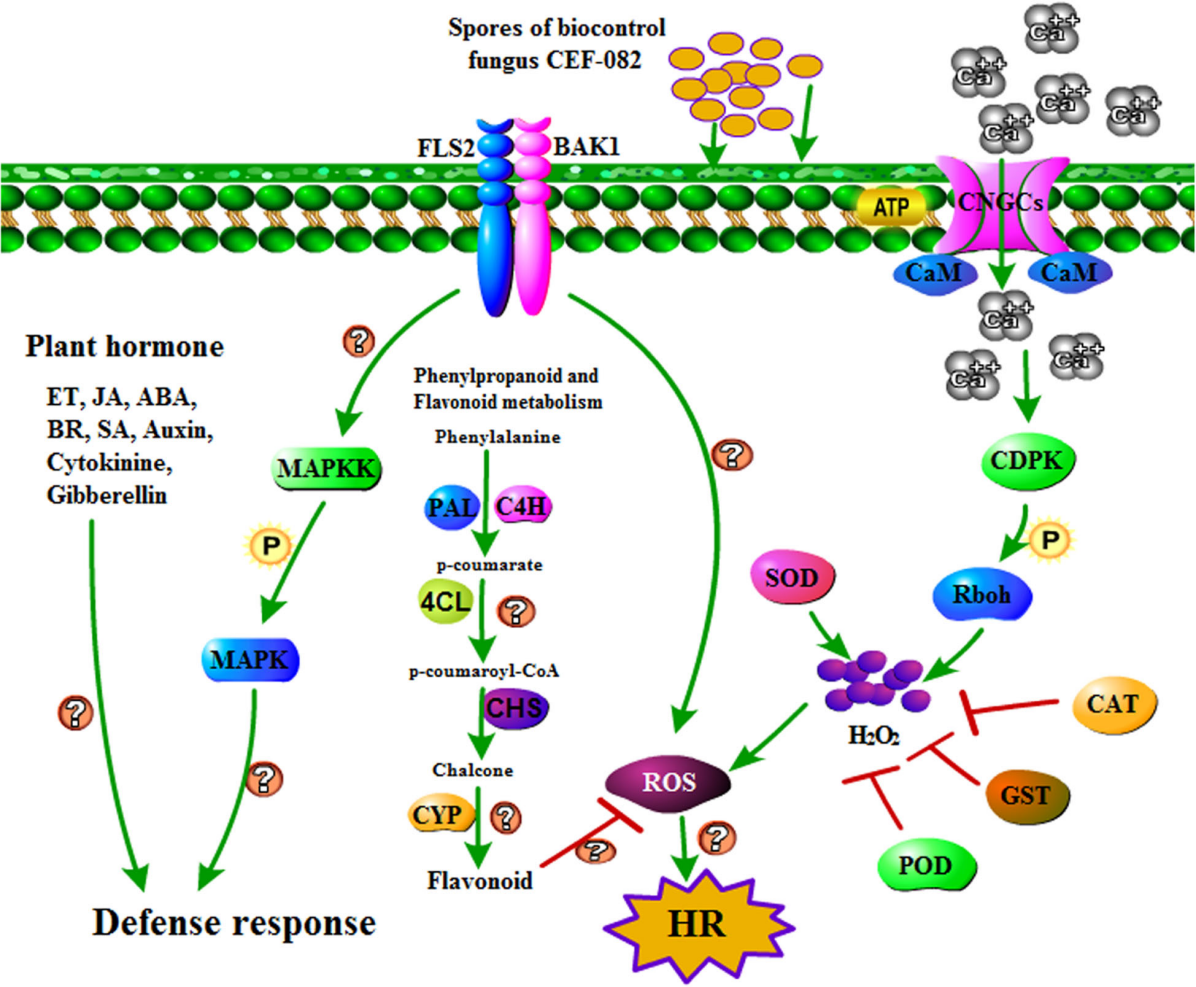
Signal transduction pathways induced by CEF-082

**Fig. 6:**
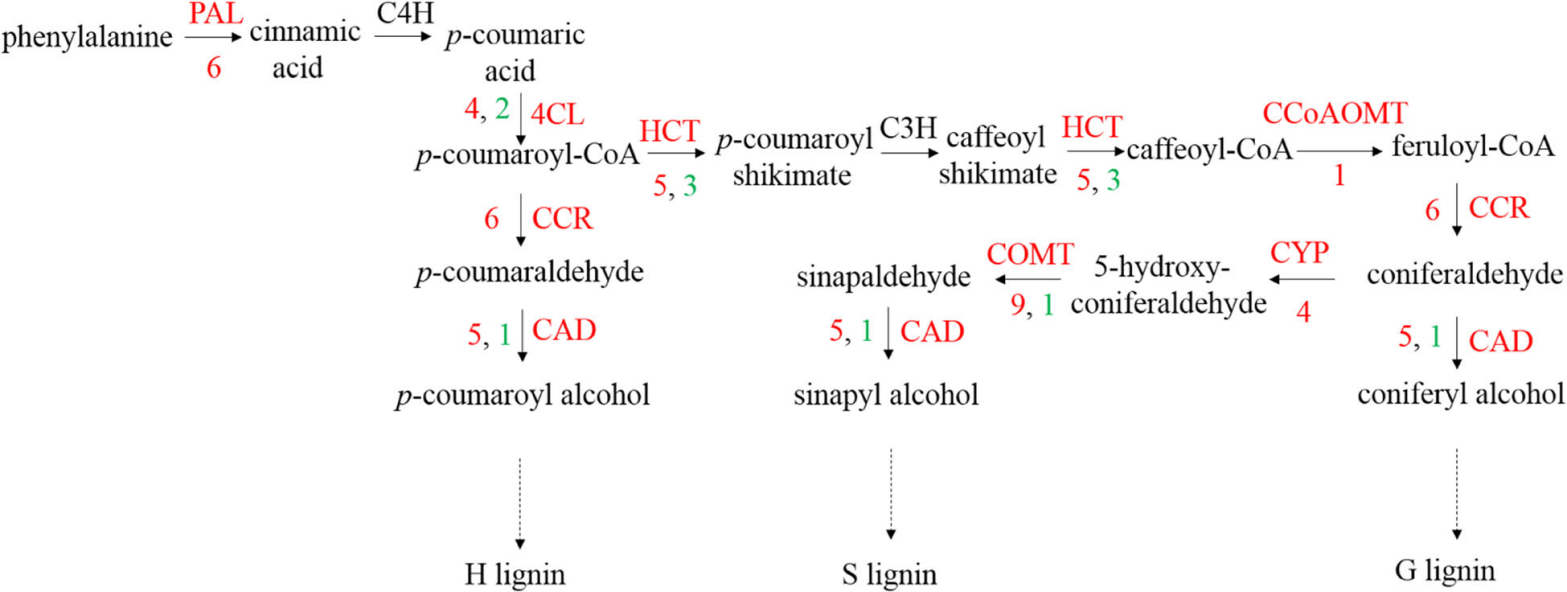
Lignin biosynthesis pathway. This figure refers to the lignin biosynthesis pathway of Miedes et al. [36]. Enzymes colored red or black indicate the key points induced or uninduced by CEF-082. The red numbers represent the number of upregulated genes, and green numbers represent the number of downregulated genes. PAL, phenylalanine ammonia-lyase; C4H, cinnamate 4-hydroxylase; 4CL, 4-coumarate-CoA ligase; C3H, *p*-coumarate 3 hydroxylase; HCT, hydroxycinnamoyl transferase; CCR, cinnamoyl CoA reductase; CAD, cinnamyl alcohol dehydrogenase; CCoAOMT, caffeoyl-CoA O-methyltransferase; F5H, ferulate-5-hydroxylase. Figure 6 is a part of the figure of Miedes E, and then added the numbers of upregulated and downregulated genes, and draw it by ourselves, not copied from the paper. The gene numbers upregulated and downregulated in Fig. 6 are our data from RNA-seq

Phenylpropane synthesis is related to cotton defense mechanisms [29], while flavonoids are known to buffer substantial stress-induced alterations in ROS homeostasis and to modulate the ROS-signalling cascade [30]. Plant CNGC subunits and CaM constitute a molecular switch that either opens or closes calcium channels [31]. Previous reports have shown that calcium-dependent CDPK4 and CDPK5 regulate ROS production by phosphorylating NADPH oxidase in potatoes [32]. ROS are important not only as defense signalling mechanisms [33] but also for regulating programmed cell death via the establishment of the HR [34]. MAPK family members can improve resistance to Verticillium wilt of cotton [35]. In this study, 24 h after CEF-082 inoculation, certain signal transduction pathways might have been involved in the plant response to CEF-082 (Fig. 5). After inoculation with CEF-082, FLS2 recognized CEF-082, MAPK signal transduction was induced, and calcium channels opened. H_2_O_2_ was then produced, leading to an ROS burst. Plant hormones were also induced, including ET, SA, JA, ABA, BR, auxin, gibberellin and cytokinin. The signalling pathways of flavonoids and phenylpropane synthesis were also involved in this process. In addition, lignin synthesis was induced after treatment with CEF-082 (Fig. 6). Figure 6 refers to the lignin biosynthesis pathway of Miedes et al. [36]. *Cinnamate 4-hydroxylase* (*C4H*) and *p-coumarate 3 hydroxylase* (*C3H*) were not induced in T0 h-vs-C0h, T12 h-vs-C12h, or T48 h-vs-C48h but were induced in C12h-vs-C0h, which was similar to the results of Xu et al. [37], who indicated that *C4H-1* and C*4H-3* were upregulated after treatment with *V. dahliae*. Three days after inoculation with *V. dahliae*, lignin was detected, and the pith diameter of CEF-082 + *V. dahliae*-treated plants was slightly larger than that of water + *V. dahliae*-treated plants (Additional file 7: Figure S7). The defense response at T12 h and T48 h was similar to that at T0 h, and only a few key points induced were different in the pathways, which are shown in Figs. 5 and 6. Thus, it is speculated that CEF-082 reduced the occurrence of cotton Verticillium wilt because inoculation with CEF-082 can prime signalling pathways involved in defense against *V. dahliae* upon infection.

When pathogens infect plants, they induce a series of defense responses. GST participates in plant defense and can remove ROS [38]. Plant GSTs can be subdivided into eight categories, phi, zeta, tau, theta, lambda, dehydroascorbate reductase (DHAR), elongation factor 1 gamma (EF1G) and tetrachlorohydroquinone dehalogenase (TCHQD) [39]. *GSTF8* has been used as a marker of early stress and defense responses [40], and JA, methyl jasmonate, ABA and H_2_O_2_ can induce *GST* expression [41–43]. *LrGSTU5* was obviously upregulated after treatment with *Fusarium oxysporum* [44], and the *GST* genes were also upregulated in *G. barbadense* treated with *V. dahliae* [45]. In this study, the *GST* genes were also significantly induced 24 h after treatment with CEF-082 (Fig. 3), and *GST* genes were upregulated in cotton treated with water + *V. dahliae*. These results are consistent with those of Han et al. and Zhang et al. [44, 45]. Certain *GST* genes were also significantly induced in the treatment group but were not significantly induced in the control group after treatment with *V. dahliae.* The *GST* gene *Gh_A09G1509* was shown to increase resistance to Verticillium wilt in tobacco [46]. Hence, we suggest that CEF-082 can induce specific *GST* genes to protect cotton from *V. dahliae*.

*V. dahliae* can induce a defense response after it infects cotton [3]. In this study, susceptible cotton varieties were inoculated with the biocontrol fungus CEF-082 and *V. dahliae*, which also induced a series of defense responses. Compared with plants inoculated with water +*V. dahliae*, the plants inoculated with CEF-082 + *V. dahliae* presented significantly upregulated or downregulated expression of resistance-related genes. Therefore, it is speculated that the defense response was strengthened after inoculation with the biocontrol fungus CEF-082. In addition, we obtained 1209 specific DEGs, that were not induced in plants inoculated with water +*V. dahliae*, but were induced only in plants inoculated with CEF-082 + *V. dahliae*. GO enrichment showed that these genes were involved in ROS metabolic process. The disease resistance of cotton was enhanced after CEF-082 treatment, and thus, we inferred that these specific DEGs might be genes related to plant disease resistance.

## Conclusion

CEF-082 can induce defense responses in cotton, and pretreatment with CEF-082 at an appropriate concentration of 10^5^spore/mL can improve the resistance of cotton (Jimian 11) to Verticillium wilt. Transcriptome analysis revealed that genes expressed in cotton leaves involved in ROS burst, Ca^2+^, lignin biosynthesis, flavonoids and phenylpropane synthesis were significantly upregulated or downregulated. Defense responses could be induced in cotton plants treated with CEF-082, and these responses were stronger in cotton plants inoculated with *V. dahliae* in the presence of CEF-082. In addition, 1209 specific DEGs induced only in plants inoculated with *V. dahliae* in the presence of the biocontrol fungus CEF-082 were obtained.

## Methods

### Fungal strain culture

The cotton endophyte *C. globosum* CEF-082 was cultured on potato dextrose agar (PDA) plates for 20 d. Spores were obtained by adding sterile water to each plate, rubbing a sterile spatula over the colony and then filtering the suspension through a sterile cheesecloth, after which the suspension was diluted to a 1 × 10^5^ spore/mL. *V. dahliae* VD1070–2 was cultured on PDA for 7 d, inoculated into liquid Czapek-Dox medium [47], and cultured in the dark at 25 °C and 150 rpm for 7 d. The mycelia were filtered out and removed, and the filtrate was subsequently diluted to a 1 × 10^7^ spore/mL spore suspension.

### Cotton inoculation treatment

Jimian 11, a highly Verticillium wilt-susceptible upland cotton variety, was provided by Professor Heqin Zhu from State Key Laboratory of Cotton Biology, Institute of Cotton Research of Chinese Academy of Agricultural Sciences. It is a cultivar selected from hybrid cross [(Jihan 4× Ke 4104) F_2_ × 74Yu102]. The seeds were sterilized with 70% alcohol for 1 min and then with 1.05% sodium hypochlorite for 10 min, after which the seeds were washed with sterile water 5 times. The cotton seeds were planted in vermiculite and transferred to plastic pots (25 cm × 15 cm) that contained 2000 mL of liquid culture solution after emergence. The cultivation solution was prepared according to the methods of Zhang et al. [48], with some modifications. In this study, 2 mM NaCl was used instead of 2.5 mM KCl, while the other 9 mineral nutrients were the same. A black foam board with 20 holes was placed on the plastic pot, and cotton plants were placed into the holes and supported by a sponge. Twenty plants were cultivated per pot per treatment, and each treatment was repeated three times. Twenty cotton plants in each treatment were removed from the plastic pots, and inoculated with CEF-082 by soaking the cotton roots in 300 mL of a 1 × 10^5^ spore/mL spore suspension for 40 min prior to the flattening of the first true leaf. For the control group, water was used instead of the CEF-082 spore suspension. The cotton plants were then returned to the pots. At 0 h, 6 h and 24 h later, 5 leaves were randomly collected at each time point for each biological replicate in each treatment, and 24 h was considered to be 0 h before inoculation with *V. dahliae* (24 h (0 h)). Twenty four hours post inoculation with CEF-082, the same method was used to inoculate *V. dahliae* VD1070–2 (1 × 10^7^ spore/mL) in the treatment group and the control group. Leaf samples were then collected at 12 h, 1 d, 2 d, 3 d, 5 d and 7 d, and 5 leaves were also randomly collected at each time point for each biological replicate under each treatment. Three biological replicates were included.

### Determination of hydrogen peroxide (H_2_O_2_) content

H_2_O_2_ content was estimated according to the methods of Sharma et al. [49] with minor modifications. Approximately 0.1 g of cotton leaves was weighed and added to 1 mL of acetone for ice bath homogenization. The samples were then centrifuged at 8000×g and 4 °C for 10 min, and the supernatant was collected. Then, 25 μL of 20% titanium chloride in concentrated HCl and 200 μL of ammonia solution (17 M) were added. The precipitate was washed 3 times with acetone. Afterward, the washed precipitates were dissolved in 1.5 mL of H_2_SO_4_ (2 N), and the absorbance was read at 415 nm.

### Control effect of the biocontrol fungus CEF-082 on Verticillium wilt of cotton

The above mentioned hydroponic seedlings were investigated at 14 d post inoculation (dpi) with VD1070–2. The disease severity was rated according to a disease index that was based on a five-scale categorization of Verticillium wilt disease of cotton seedlings [50].

### RNA sequencing (RNA-seq)

A polysaccharide polyphenol RNA extraction kit (TianGen, Beijing) was used to extract RNA from cotton leaves. Electrophoresis was performed, and a One Drop (1000+) spectrophotometer was used to detect the concentration and quality of RNA. Transcriptome sequencing was performed for the 24 h (0 h (T0 h, C0h)), 12 h (T12 h, C12h) and 48 h (T48 h, C48h) samples. T0 h, T12 h and T48 h represented the 0, 12 and 48 h samples in the treatment group, respectively, and C0h, C12h and C48h represented the 0, 12 and 48 h samples in the control group, respectively. Three biological replicates were performed, and there were 18 samples. The construction of the DNA library and sequencing were performed by Beijing Genomics Institute (BGI). Data filtering was performed using SOAPnuke software (BGI, Beijing). Clean reads were obtained by removing the reads containing adapters, reads with more than 5% N, and low-quality sequences. The clean reads were spliced and aligned to the reference *G. hirsutum* genome retrieved from the cotton genome website (https://www.cottongen.org/). The fragments per kilobase per transcript per million mapped reads (FPKM) values were calculated and used to estimate the effects of sequencing depth and gene length on the mapped read counts.

### Screening and analysis of differentially expressed genes (DEGs)

The DEGseq R package (1.20.0) [51] was used to analyze DEGs in cotton leaves treated or nontreated with CEF-082 under the criteria of a corrected *P* value < 0.001 and an absolute log2 ratio ≥ 1. GO (Gene Ontology) terms and KEGG (Kyoto Encylopedia of Genes and Genomes) pathways were enriched by DEGs if the *P* values were < 0.001. Resistance genes among the DEGs were predicted by a BLAST search of the Plant Resistance Gene (PRG) Database (identity ≥40, *E*-value <1E-5) [52]. TFs encoded by the DEGs were predicted (*E*-value <1E-5) according to the Plant Transcription Factor Database [53].

### Quantitative reverse-transcription-PCR (qRT-PCR) analysis

The plant-pathogen interaction pathway and R genes are important for plant resistance. Twelve DEGs involved in the plant-pathogen interaction pathway and predicted R genes were randomly selected for qRT-PCR to verify whether the trends in their expression was consistent with the transcriptome sequencing results. Data were collected from three replicate experiments, and the samples used for qRT-PCR were the same as those used for RNA-seq. RNA was extracted from sample leaves and reverse transcribed into cDNA. qRT-PCR was performed via a Bio-Rad CFX96 Real-Time System (Bio-Rad, USA), and each PCR mixture (20 μL) consisted of 10 μL SuperReal PreMix Plus SYBR Green (Tiangen), 0.4 μL of each primer, 2 μL of cDNA and 7.2 μL of sterile water. Each sample involved at least three technical repeats. The PCR cycle consisted of an initial denaturation step of 95 °C for 10 min, followed by 40 cycles of 95 °C for 30 s, 60 °C for 30 s and 72 °C for 30 s. The cotton *ubiquitin* gene was used as the internal reference, and relative gene expression was calculated using the 2^-ΔCT^ method. Primers were obtained from the upland cotton gene fluorescence quantitative specific primer database (https://biodb.swu.edu.cn/qprimerdb/) (Additional file 8: Table S1).

## Supplementary information

**Additional file 1: Figure S1.** Correlation thermograms of the 18 samples.

**Additional file 2: Figure S2.** PCA of the 18 samples.

**Additional file 3: Figure S3.** Clustering thermogram of 1209 DEGs.

**Additional file 4: Figure S4.** GO enrichment analysis of 1209 DEGs.

**Additional file 5: Figure S5.** Venn diagram of DEGs.

**Additional file 6: Figure S6.** Protein interaction network of 96 DEGs and their related genes in cotton. The red font indicates hub genes.

**Additional file 7: Figure S7.** Histochemical analysis of lignin in stem cross-sections of cotton plants.

**Additional file 8: Table S1.** Specific primer sequences used for qRT-PCR.

**Additional file 9: Table S2.** Sequencing quality statistics table.

**Additional file 10: Table S3.** Overview of novel transcripts.

**Additional file 11: Table S4.** Putative R genes and genes encoding TFs among the 96 DEGs.

**Additional file 12.** Data concerning the H_2_O_2_ content.

**Additional file 13.** Data concerning the disease index.

**Additional file 14.** qPCR data.

## Data Availability

Most data supporting the results are included in the article and additional files (Additional files 12, 13 and 14). Other data are available from the corresponding authors on reasonable request.

## Abbreviations

4CL: 4-coumarate-CoA ligase
ABA: Abscisic acid
BR: Brassinosteroid
C3H: P-coumarate 3 hydroxylase
C4H: Cinnamate 4-hydroxylase
CAD: Cinnamyl alcohol dehydrogenase
CAT: Catalase
CCoAOMT: Caffeoyl-CoA O-methyltransferase
CDPK: Calcium-dependent protein kinase
CHS: Chalcone synthase
CML: Calcium-binding protein
COMT: Caffeoyl O-methyltransferase
CYP: Cytochrome P450 proteins
ERF: Ethylene-responsive transcription factor
ET: Ethylene
F5H: Ferulate-5-hydroxylase
FLS2: Flagellin-sensitive 2
GST: Glutathione S-transferase
HR: Hypersensitive response
HSP90: Heat shock protein 90 kDa
JA: Jasmonic acid
PAL: Phenylalanine ammonia-lyase
POD: Peroxidase
Pti1: Pto-interacting protein 1
qRT-PCR: Quantitative reverse-transcription-PCR
Rboh: Respiratory burst oxidase homologue
ROS: Reactive oxygen species
RPS2: Disease resistance protein RPS2
SA: Salicylic acid
SOD: Superoxide dismutase
