# Supplementary figures and images for: Transcriptome analysis reveals the defense mechanism of cotton against Verticillium dahliae in the presence of the biocontrol fungus Chaetomium globosum CEF-082
Source: BMC Plant Biol. 2020 Feb 27;20:89. doi: 10.1186/s12870-019-2221-0 (PMC7047391; doi:10.1186/s12870-019-2221-0)

**Figure S1**


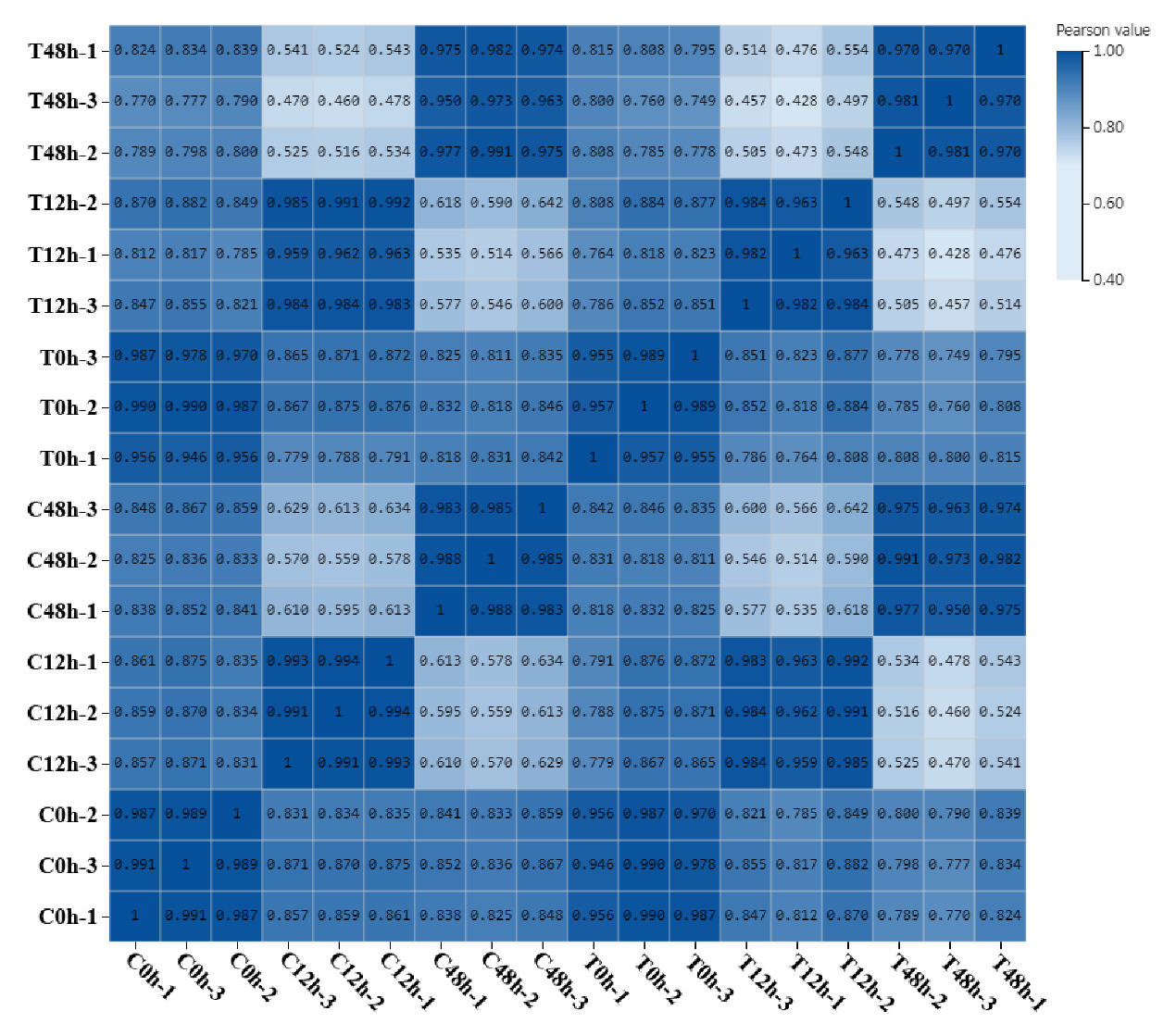

Supplement: Supplementary file 1 — Additional file 1: Figure S1. Correlation thermograms of the 18 samples. [file 12870_2019_2221_MOESM1_ESM.docx]

**Figure S2**

**
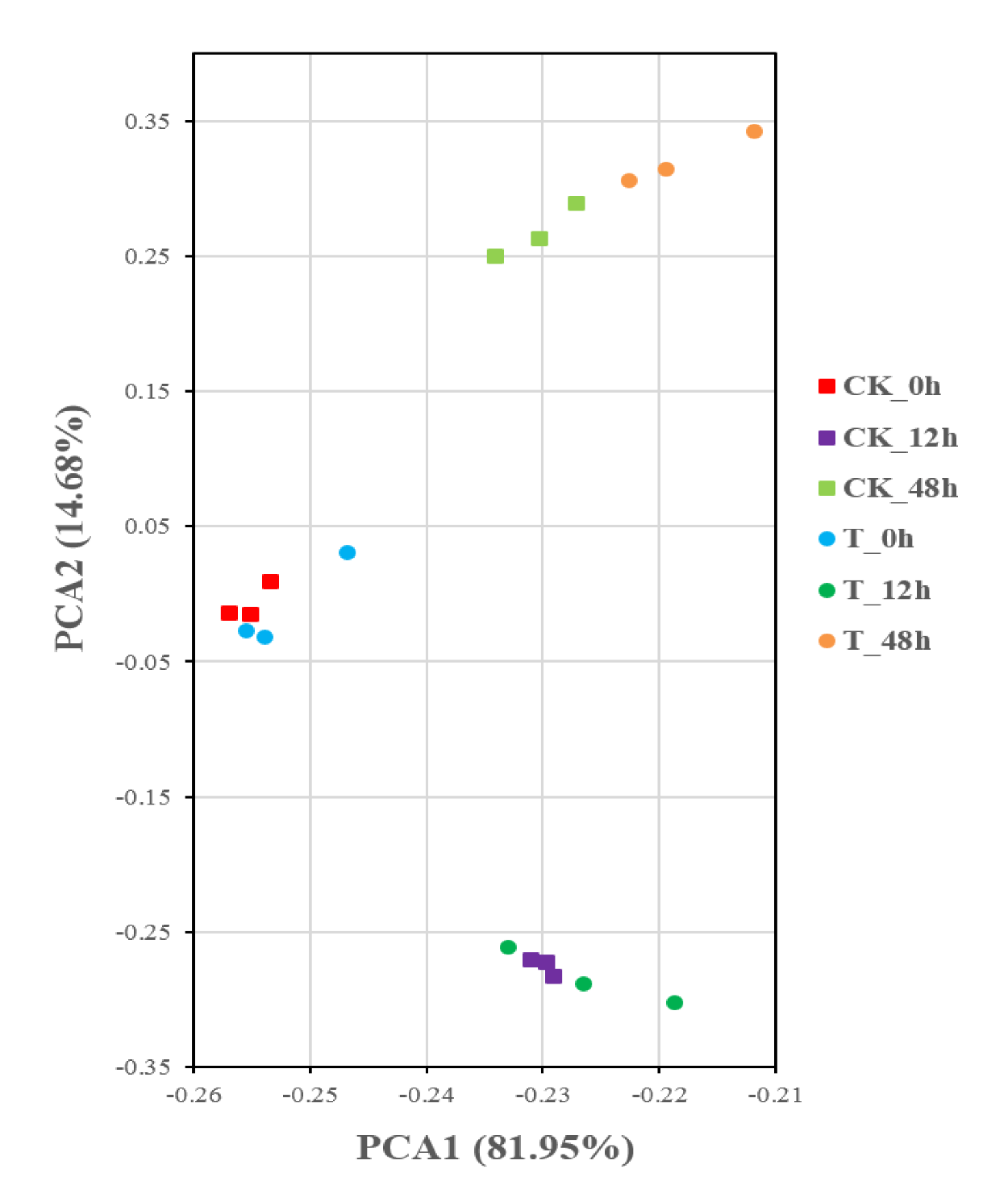
**

Supplement: Supplementary file 2 — Additional file 2: Figure S2. PCA of the 18 samples. [file 12870_2019_2221_MOESM2_ESM.docx]

**Figure S3**


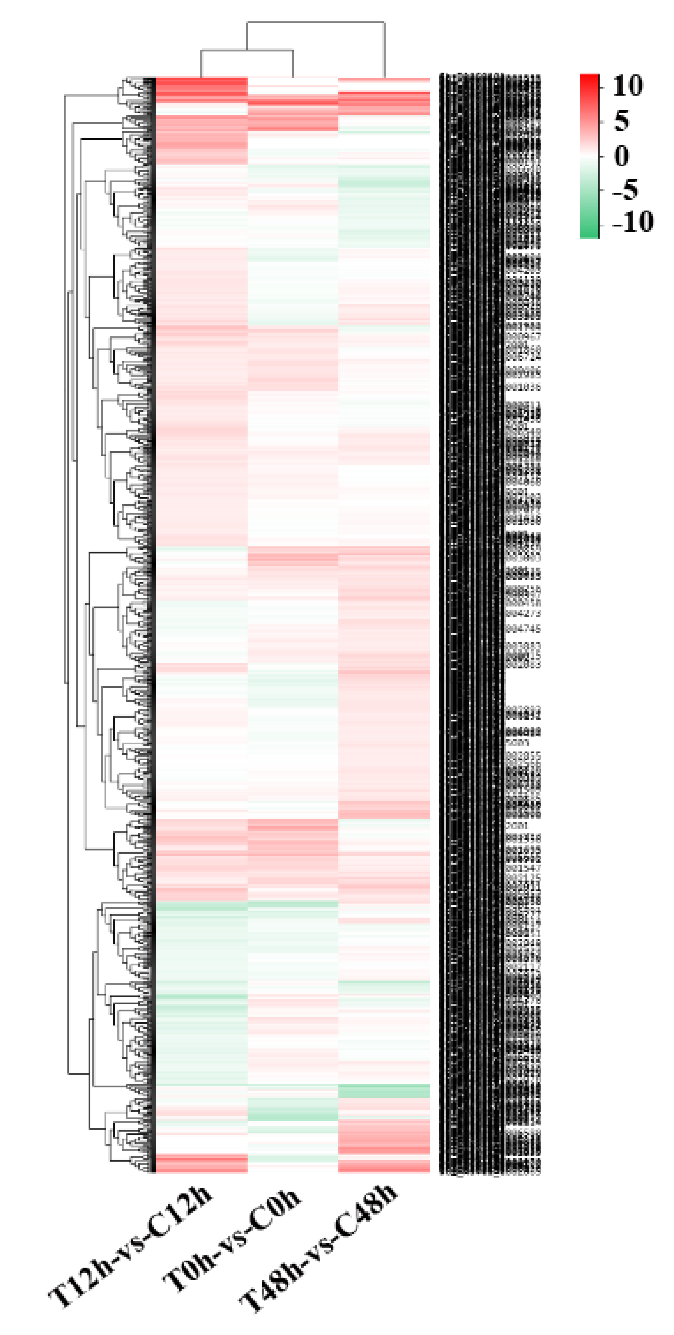

Supplement: Supplementary file 3 — Additional file 3: Figure S3. Clustering thermogram of 1209 DEGs. [file 12870_2019_2221_MOESM3_ESM.docx]

**Figure S4**


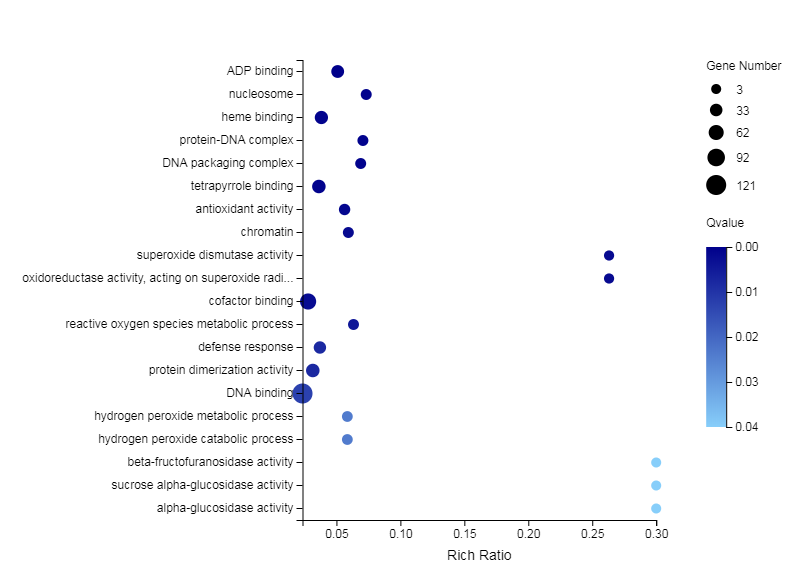

Supplement: Supplementary file 4 — Additional file 4: Figure S4. GO enrichment analysis of 1209 DEGs. [file 12870_2019_2221_MOESM4_ESM.docx]

**Figure S5**

**
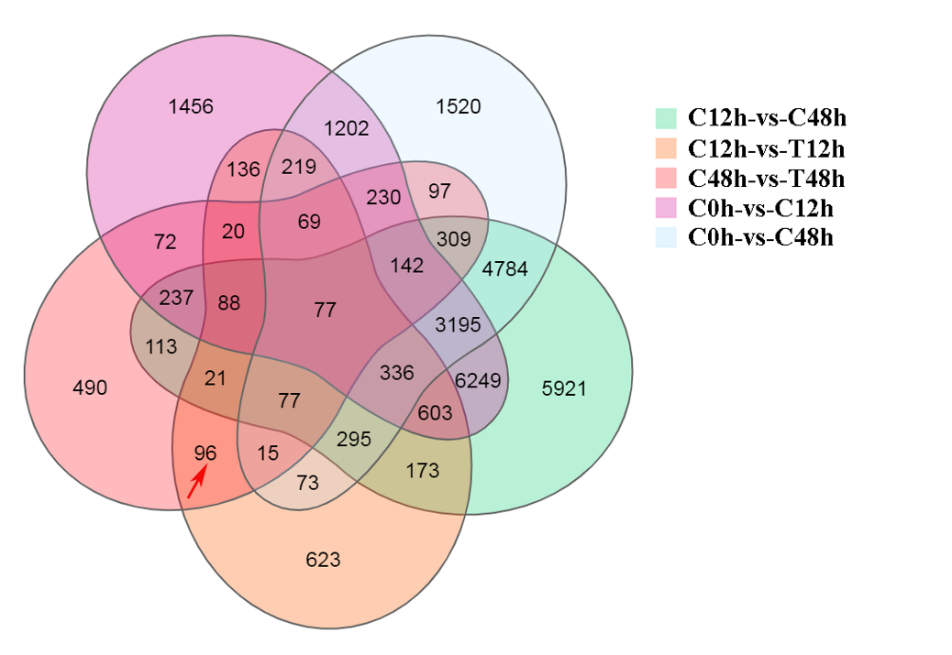
**

Supplement: Supplementary file 5 — Additional file 5: Figure S5. Venn diagram of DEGs. [file 12870_2019_2221_MOESM5_ESM.docx]

**Figure S6**

**
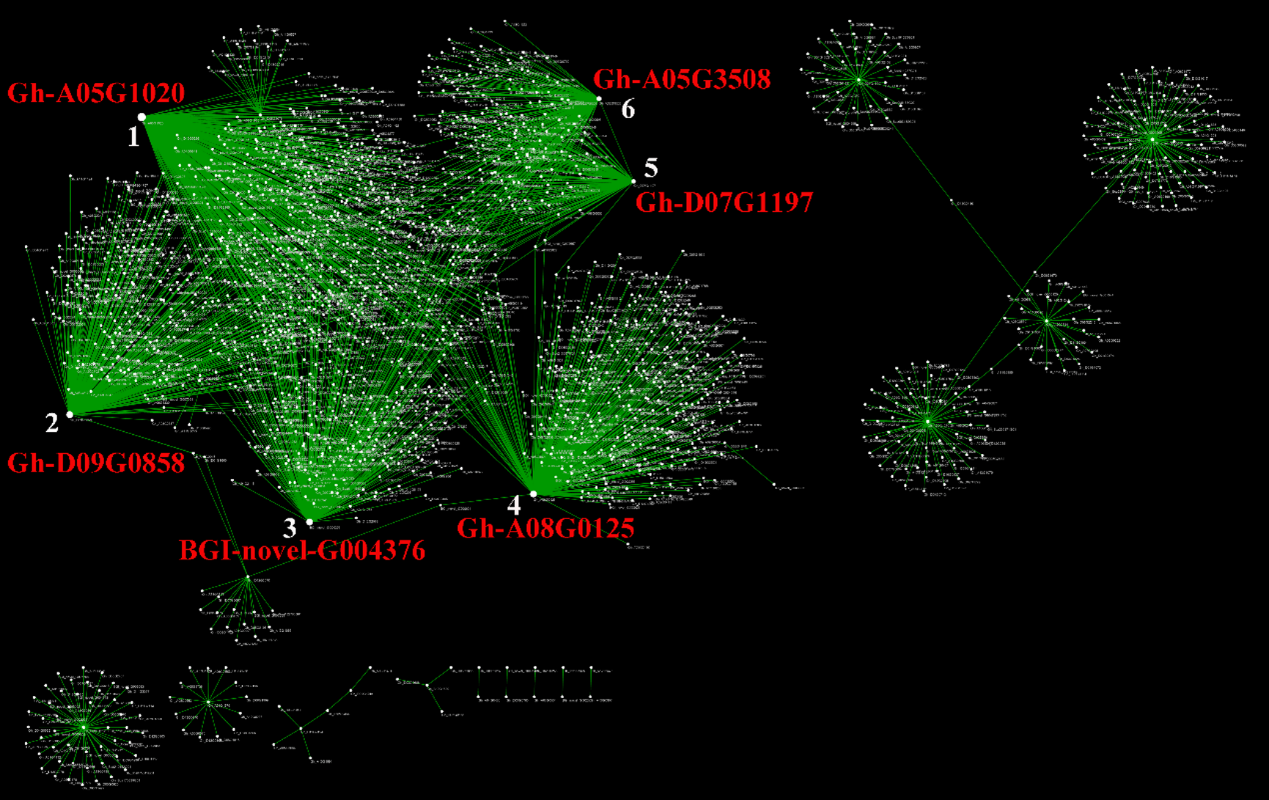
**

Supplement: Supplementary file 6 — Additional file 6: Figure S6. Protein interaction network of 96 DEGs and their related genes in cotton. The red font indicates hub genes. [file 12870_2019_2221_MOESM6_ESM.docx]

**Figure S7**


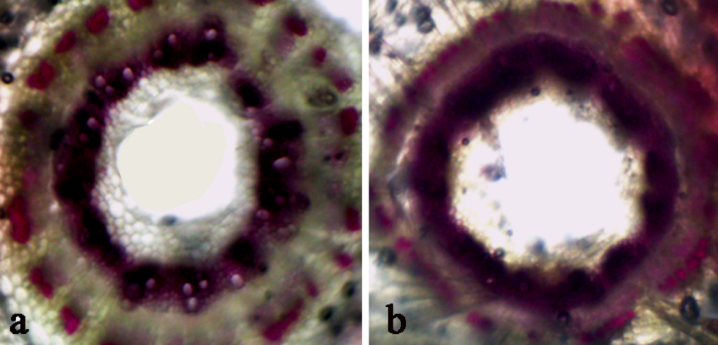

Supplement: Supplementary file 7 — Additional file 7: Figure S7. Histochemical analysis of lignin in stem cross-sections of cotton plants. [file 12870_2019_2221_MOESM7_ESM.docx]
