# Supplementary material for: Transcriptome analysis reveals the defense mechanism of cotton against Verticillium dahliae in the presence of the biocontrol fungus Chaetomium globosum CEF-082
Source: BMC Plant Biol. 2020 Feb 27;20:89. doi: 10.1186/s12870-019-2221-0 (PMC7047391; doi:10.1186/s12870-019-2221-0)
Supplement: Supplementary file 8 — Additional file 8: Table S1. Specific primer sequences used for qRT-PCR. [file 12870_2019_2221_MOESM8_ESM.docx]

**Table S1** Specific primer sequences used for qRT-PCR

| Gene name | Primer sequence (5′→3′) | |
| --- | --- | --- |
| *Gh_D11G1515* | F: AGTTGGTCTCTCAGGGGTGGAG; | R: ACACATAGCAGAACGGTGGCTC |
| *Gh_D11G1887* | F: GTTAAGCCTTCACCGTCGTGGA; | R: TGTACTCCCCGATGGACCCAAA |
| *Gh_D08G2484* | F: AAGGACTGCCTTCTTGTTGGGG; | R: GCTCACGTTTTCTAGGCCGCTA |
| *Gh_D05G0689* | F: GCTTTGTTGAGCGGCAACATGA; | R: AGGAGGAATCACCCCCGACATT |
| *Gh_A05G0560* | F: GCCGACAACCAATTATCGGGGA; | R: ACTGAGATTCCCGGCTTTGCTG |
| *Gh_A05G0483* | F: GCCGAGTGTGAGGATTATGCCA; | R: TTCTTGACGGGACAACTTGGGG |
| *Gh_A04G0855* | F: TCTGCAGCTCACGTCAGCATTT; | R: GCGGAAGAAGCTTCACGGTTTC |
| *Gh_D05G2642* | F: GCAGCAACCCCAAAATCCCAAC; | R: CCACATCCCCGTATTCAGCACC |
| *Gh_D05G3615* | F: CGGCTCAACAACAATTCCAGGC; | R: AAGGCACAACATCATGGGGCAT |
| *Gh_D07G0457* | F: GTCCGCTGCAGCTACTCAGTTT; | R: CCCAAACAAGGGGATTCACAGC |
| *Gh_D12G2793* | F: GAAAGTGAAAGCGATGCCTGCC; | R: CTGCTTGCCTGTTTCCATGTCG |
| *Gh_D06G1832* | F: TATCATGGCACATTTGCCGCCT; | R: TACGCAAGCAGGAATAGGACCG |
| *ubiquitin* | F: GAGTCTTCGGACACCATTG; | R: CTTGACCTTCTTCTTCTTGTGC |
