# Supplementary material for: Transcriptome analysis reveals the defense mechanism of cotton against Verticillium dahliae in the presence of the biocontrol fungus Chaetomium globosum CEF-082
Source: BMC Plant Biol. 2020 Feb 27;20:89. doi: 10.1186/s12870-019-2221-0 (PMC7047391; doi:10.1186/s12870-019-2221-0)
Supplement: Supplementary file 9 — Additional file 9: Table S2. Sequencing quality statistics table. [file 12870_2019_2221_MOESM9_ESM.docx]

**Table S2** Sequencing quality statistics table

| Sample | Total Raw Reads (M) | Total Clean Reads (M) | Total Clean Bases (Gb) | Clean Reads Q20 (%) | Clean Reads Q30 (%) | Clean Reads Ratio (%) |
| --- | --- | --- | --- | --- | --- | --- |
| C0h-1 | 64.46 | 62.05 | 6.21 | 98.25 | 91.1 | 96.27 |
| C0h-2 | 64.45 | 61.72 | 6.17 | 98.75 | 93.25 | 95.77 |
| C0h-3 | 64.5 | 61.79 | 6.18 | 98.68 | 92.79 | 95.8 |
| C12h-1 | 63.57 | 60.89 | 6.09 | 98.65 | 92.44 | 95.8 |
| C12h-2 | 67.41 | 64.57 | 6.46 | 98.66 | 92.36 | 95.78 |
| C12h-3 | 62.83 | 60.09 | 6.01 | 98.65 | 92.34 | 95.64 |
| C48h-1 | 65.89 | 63.17 | 6.32 | 98.65 | 93.06 | 95.87 |
| C48h-2 | 63.6 | 61.08 | 6.11 | 98.24 | 91.33 | 96.03 |
| C48h-3 | 64.63 | 62.17 | 6.22 | 98.58 | 92.71 | 96.19 |
| T0h-1 | 65.73 | 62.72 | 6.27 | 98.56 | 92 | 95.42 |
| T0h-2 | 62.92 | 60.16 | 6.02 | 98.67 | 92.44 | 95.61 |
| T0h-3 | 63.53 | 60.84 | 6.08 | 98.59 | 92.13 | 95.76 |
| T12h-1 | 68.67 | 65.48 | 6.55 | 98.61 | 92.18 | 95.35 |
| T12h-2 | 63.74 | 60.97 | 6.1 | 98.5 | 92.1 | 95.65 |
| T12h-3 | 63.51 | 61.17 | 6.12 | 98.66 | 93.11 | 96.32 |
| T48h-1 | 64.5 | 62.4 | 6.24 | 97.93 | 90.06 | 96.74 |
| T48h-2 | 65.31 | 63.1 | 6.31 | 98.56 | 92.46 | 96.62 |
| T48h-3 | 65.99 | 63.02 | 6.3 | 98.53 | 92.37 | 95.5 |
