# Supplementary material for: Transcriptome analysis reveals the defense mechanism of cotton against Verticillium dahliae in the presence of the biocontrol fungus Chaetomium globosum CEF-082
Source: BMC Plant Biol. 2020 Feb 27;20:89. doi: 10.1186/s12870-019-2221-0 (PMC7047391; doi:10.1186/s12870-019-2221-0)
Supplement: Supplementary file 10 — Additional file 10: Table S3. Overview of novel transcripts. [file 12870_2019_2221_MOESM10_ESM.docx]

**Table S3** Overview of novel transcripts

| Total Novel Transcripts | Coding Transcripts | Noncoding Transcripts | Novel Isoform | Novel Gene |
| --- | --- | --- | --- | --- |
| 47183 | 37827 | 9356 | 30539 | 7288 |
