# Supplementary material for: Transcriptome analysis reveals the defense mechanism of cotton against Verticillium dahliae in the presence of the biocontrol fungus Chaetomium globosum CEF-082
Source: BMC Plant Biol. 2020 Feb 27;20:89. doi: 10.1186/s12870-019-2221-0 (PMC7047391; doi:10.1186/s12870-019-2221-0)
Supplement: Supplementary file 11 — Additional file 11: Table S4. Putative R genes and genes encoding TFs among the 96 DEGs. [file 12870_2019_2221_MOESM11_ESM.docx]

**Table S4** Putative R genes and genes encoding TFs among the 96 DEGs.

| Gene ID | Type | Family | Gene ID | Type | Nr |
| --- | --- | --- | --- | --- | --- |
| Gh_A06G1144 | TF | AP2-EREB | BGI_novel_G000200 | PRG | / |
| Gh_A11G2091 | TF | MYB | BGI_novel_G000358 | PRG | / |
| Gh_A12G1620 | TF | NAC | BGI_novel_G000414 | PRG | / |
| Gh_D09G1659 | TF | MYB | BGI_novel_G000458 | PRG | / |
| Gh_D13G0346 | TF | bHLH | BGI_novel_G001573 | PRG | / |
| Gh_D09G1346 | TF | PLATZ | BGI_novel_G003923 | PRG | / |
| Gh_A09G2473 | TF | C2H2 | BGI_novel_G006536 | PRG | / |
| Gh_D12G2494 | TF | AP2-EREBP | Gh_A01G0470 | PRG | putative RING-H2 finger protein ATL19 [*Gossypium hirsutum*] |
| Gh_A13G0307 | TF | bHLH | Gh_A04G0855 | PRG | uncharacterized protein LOC108457923 isoform X2 [*Gossypium arboreum*] |
|  |  |  | Gh_A06G1144 | PRG | ethylene-responsive transcription factor 4-like [*Gossypium hirsutum*] |
|  |  |  | Gh_A11G2091 | PRG | transcription repressor MYB5-like [*Gossypium arboreum*] |
|  |  |  | Gh_A12G1620 | PRG | NAC domain-containing protein 100-like [*Gossypium hirsutum*] |
|  |  |  | Gh_D01G1550 | PRG | lipase [*Corchorus capsularis*] |
|  |  |  | Gh_D05G3615 | PRG | hypothetical protein B456_009G443300 [*Gossypium raimondii*] |
|  |  |  | Gh_D08G1656 | PRG | MOB kinase activator-like 1A isoform X1 [*Gossypium hirsutum*] |
|  |  |  | Gh_D09G1659 | PRG | MYB-related protein 308-like [*Gossypium hirsutum*] |
|  |  |  | Gh_D09G1718 | PRG | uncharacterized protein LOC105800125 [*Gossypium raimondii*] |
|  |  |  | Gh_D11G0790 | PRG | putative casein kinase II subunit beta-4 [*Gossypium hirsutum*] |
|  |  |  | Gh_D12G2494 | PRG | putative dehydration responsive element binding protein [*Gossypium hirsutum*] |
|  |  |  | Gh_Sca089655G01 | PRG | uncharacterized protein LOC107949870 [*Gossypium hirsutum*] |
